# Supplementary material for: Diagnostic and commensal Staphylococcus pseudintermedius genomes reveal niche adaptation through parallel selection of defense mechanisms
Source: Nat Commun. 2023 Nov 3;14:7065. doi: 10.1038/s41467-023-42694-5 (PMC10624692; doi:10.1038/s41467-023-42694-5)
Supplement: Supplementary file 1 — Supplementary Information [file 41467_2023_42694_MOESM1_ESM.pdf]

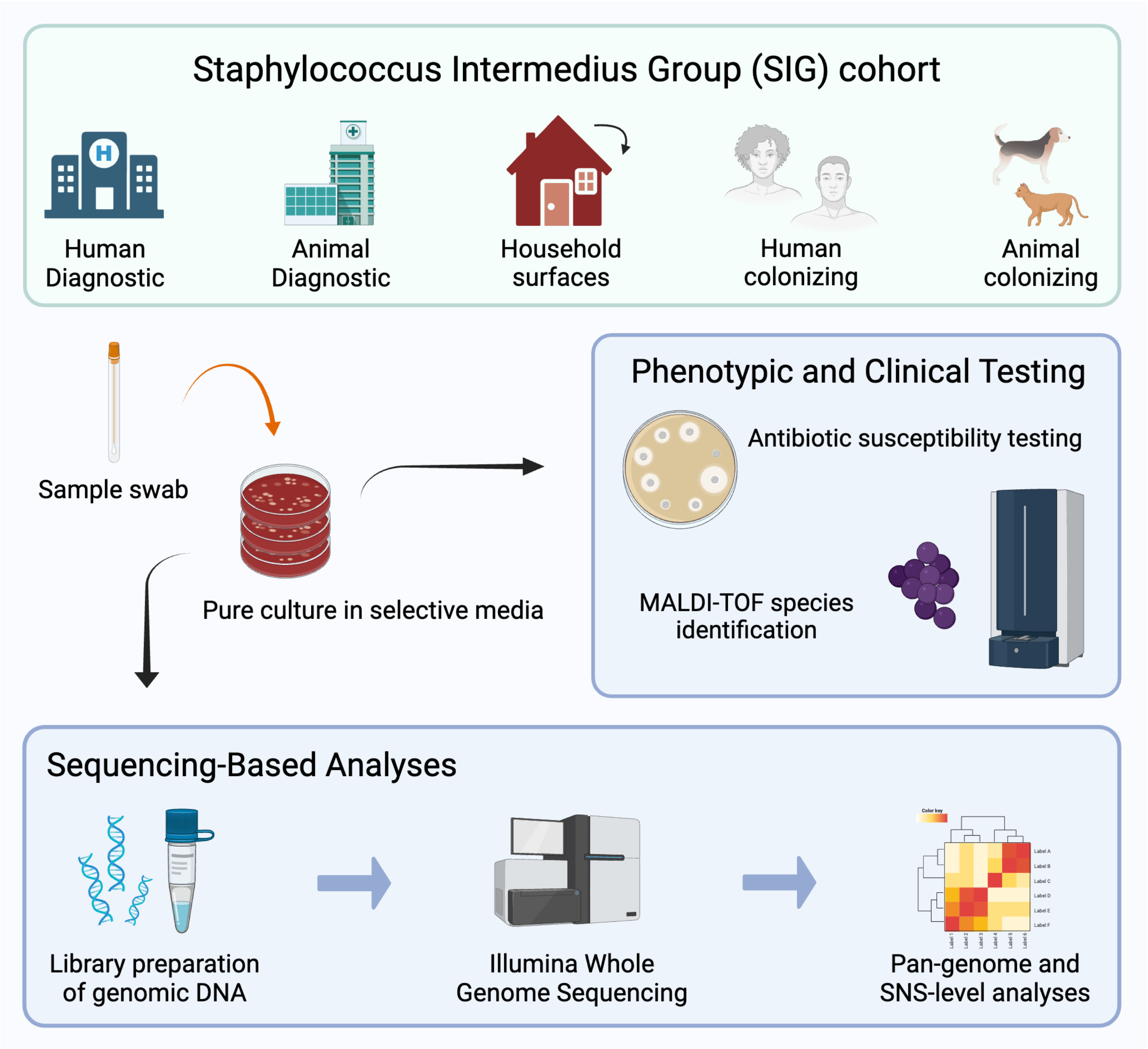

Supplementary Figure 1: Workflow schematic. Isolates from human diagnostic, animal diagnostic, and household/environment cohorts undergo phenotypic and clinical testing, along with DNA extraction, sequencing, and comparative genomic analyses. Created with Biorender.com.

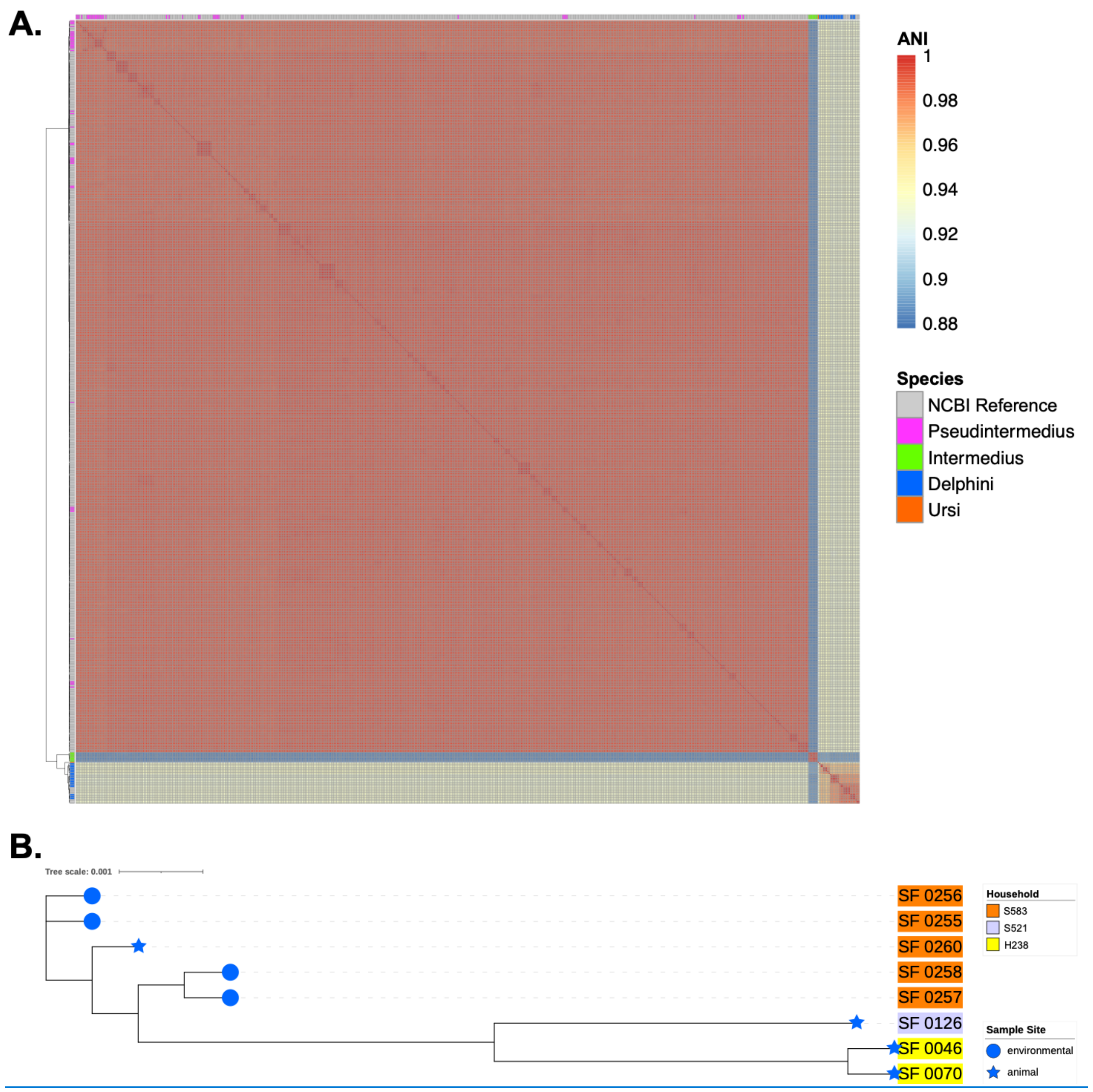

Supplementary Figure 2: WGS identifies 493 isolates as *S. pseudintermedius* and 8 as *S. delphini*. (A) Pairwise ANI of 500 sequenced isolates with 70 NCBI reference assemblies. The large upper-left quadrant represents *S. pseudintermedius* assemblies. The next compact quadrant is composed of *S. intermedius* assemblies, all of which originated from NCBI. The bottom-right quadrant represents a mix of *S. delphini* isolates from our cohort and from NCBI. **(B)** Core genome phylogeny of the eight *S. delphini* isolates captured in our household cohort.

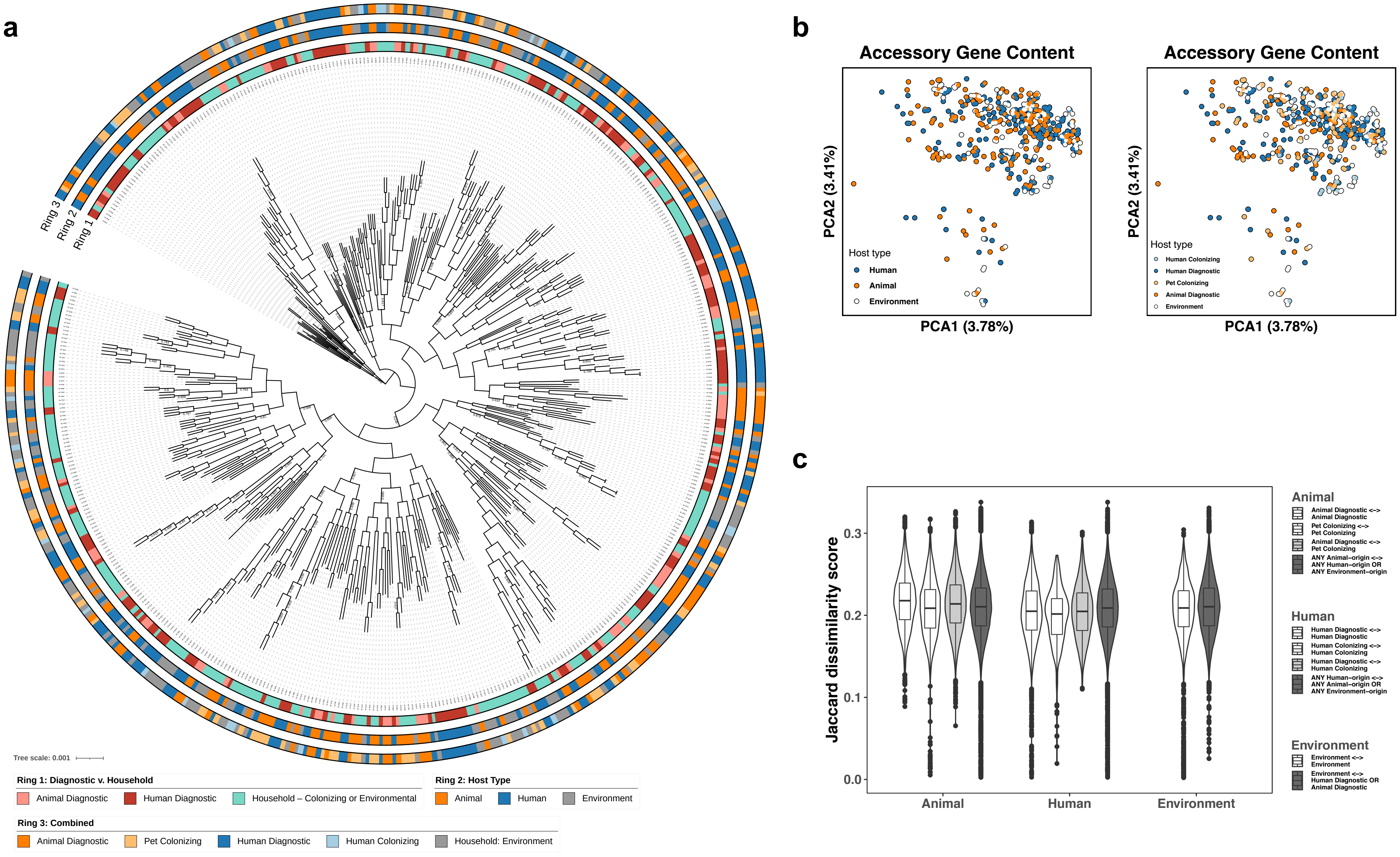

Supplementary Figure 3: Host-species adaptation is not apparent via total encoded gene architecture. (A) Core gene phylogeny of the 493 *S. pseudintermedius* isolates. Ring 1 represents niche-type consistent with Figure 1A. Rings 2-3 describe host-species or environment for each isolate. **(B-C)** Jaccard dissimilarity by accessory gene content similarity represented by **(B)** principal coordinate analysis ordination and **(C)** beta diversity compositions of between-group and within-group Jaccard distance. White, light gray, and dark gray violins indicate within host-species and niche, within host-species only, and across host-species and niches, respectively.

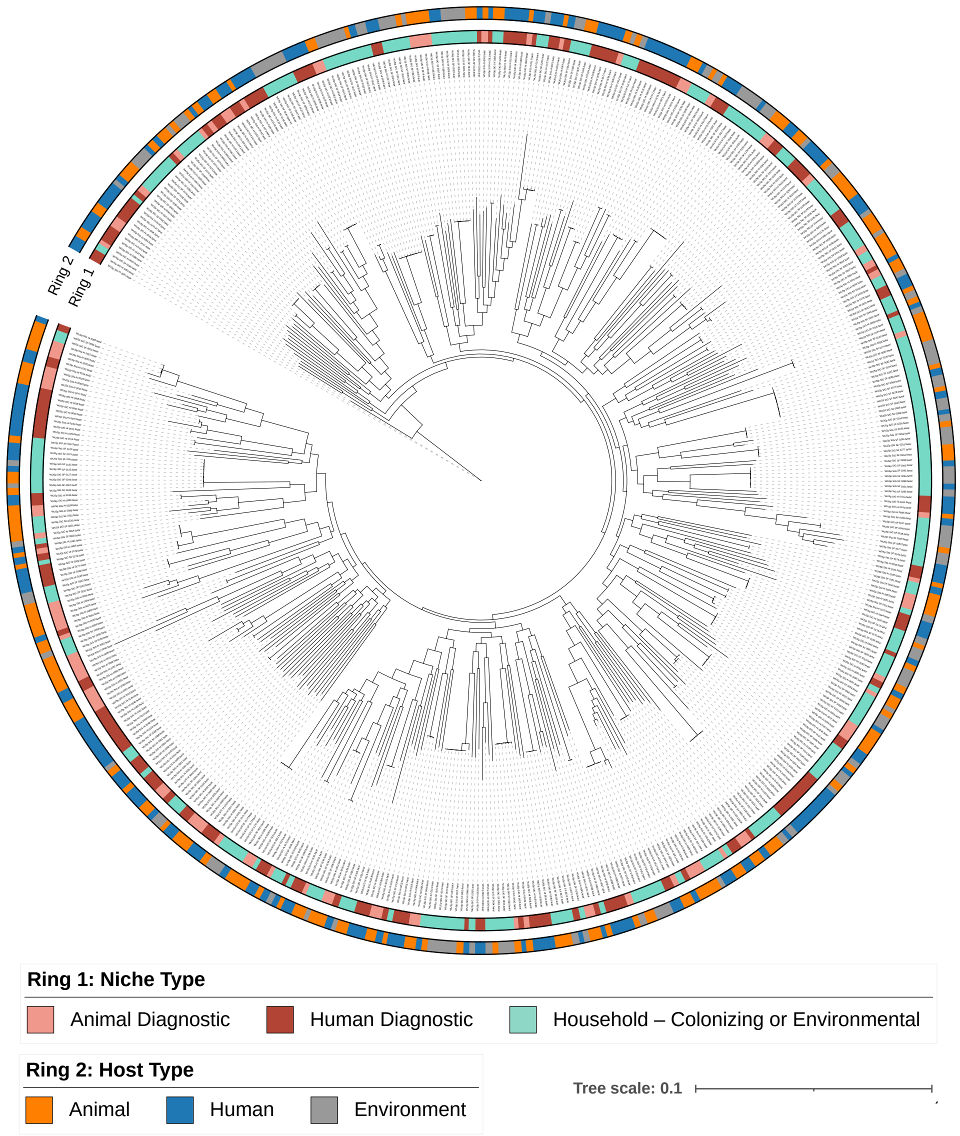

Supplementary Figure 4: Core genome phylogeny similarly reflects lack of clustering by niche type and host-species as determined by core gene phylogeny. Core genome phylogeny of the 493 *S. pseudintermedius* isolates. Ring 1 represents niche-type consistent with Figure 1A. Rings 2 represent host-species, consistent with Supplementary Figure 3A.

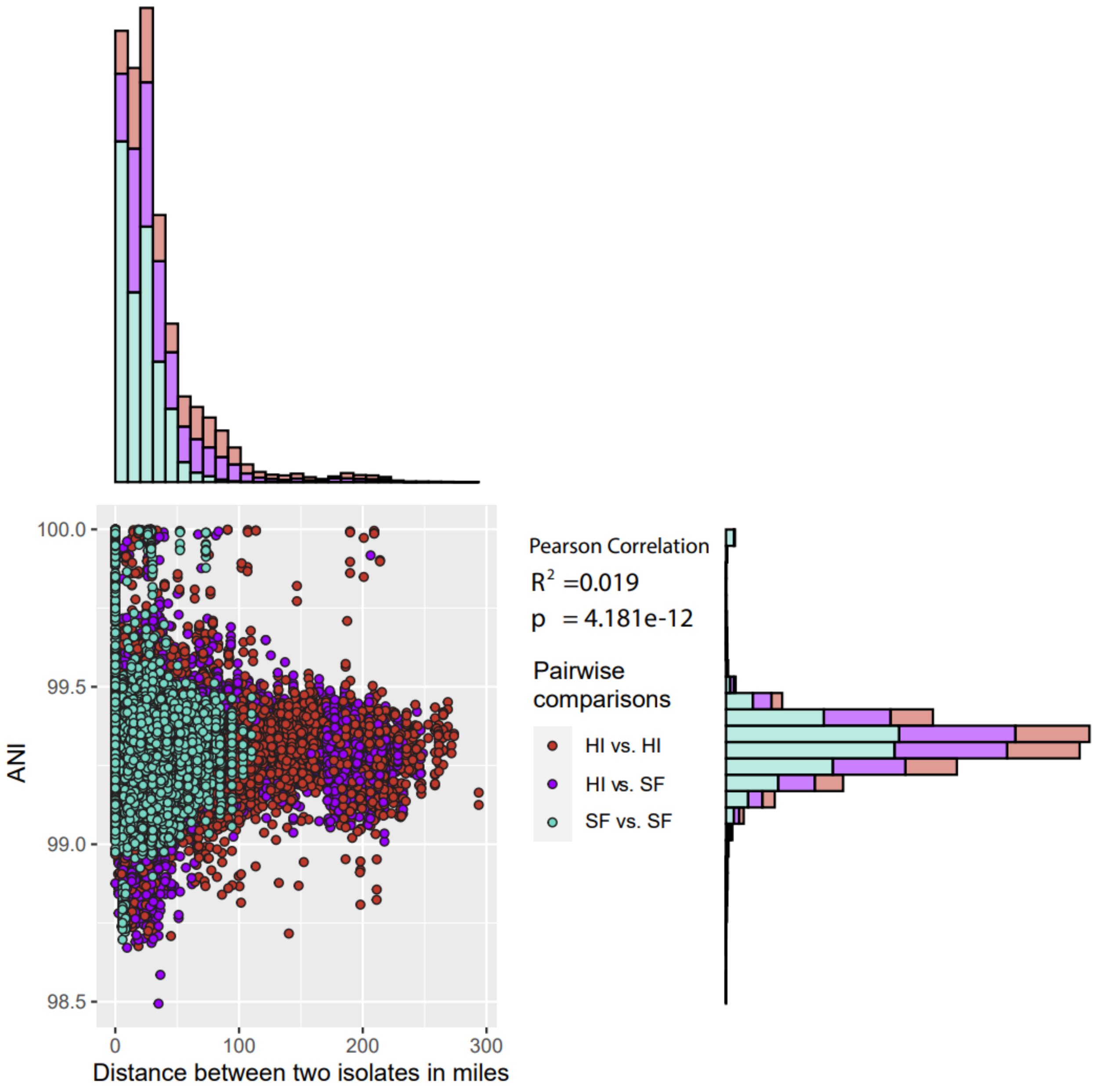

Supplementary Figure 5: Relationship between ANI and geographic distance. Self-comparisons were removed. Animal diagnostic isolates were excluded based on availability of zip code data. The centroid of each zipcode was used to calculate the latitude and longitude for each isolate. HI and SF refer to human diagnostic and environmental isolates, respectively.

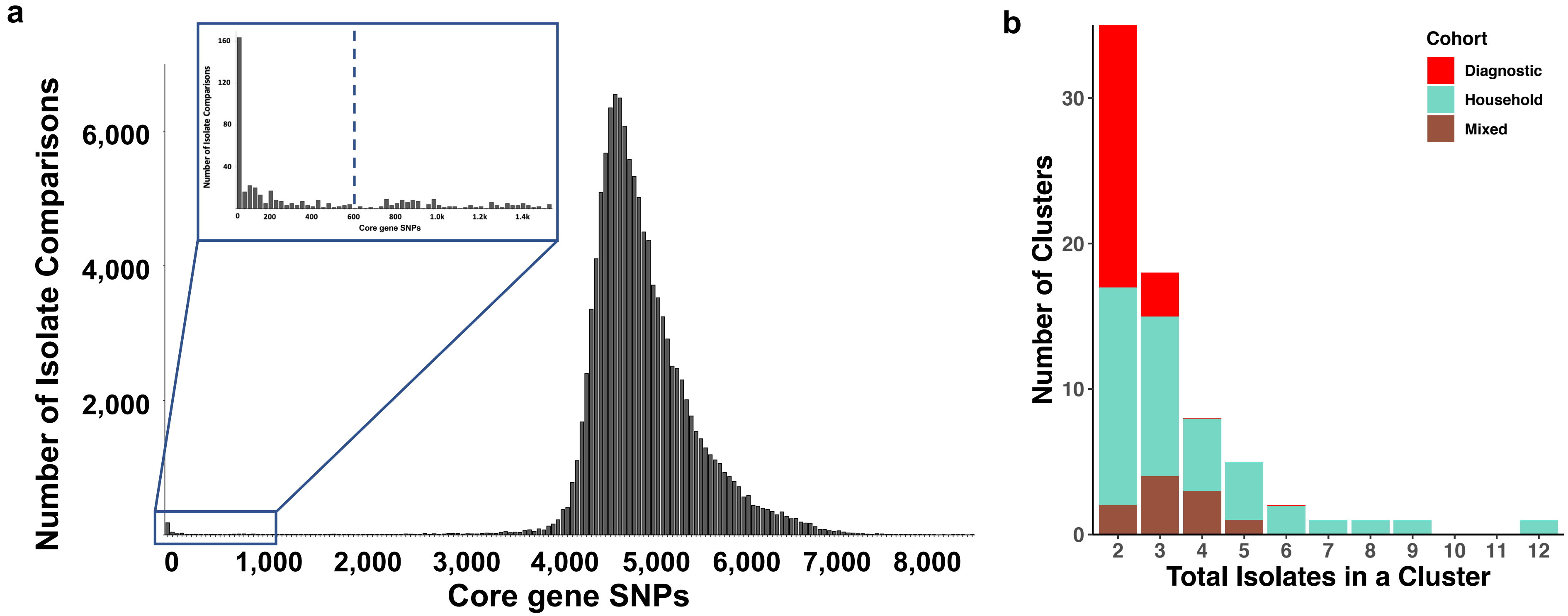

Supplementary Figure 6: Determination of lineage cluster boundaries. (A) Pairwise core gene SNP distance. 600 core gene SNPs (inlet) was the designated cutoff for lineage relationships. **(B)** Cohort composition of lineage clusters, plotted by cluster size.

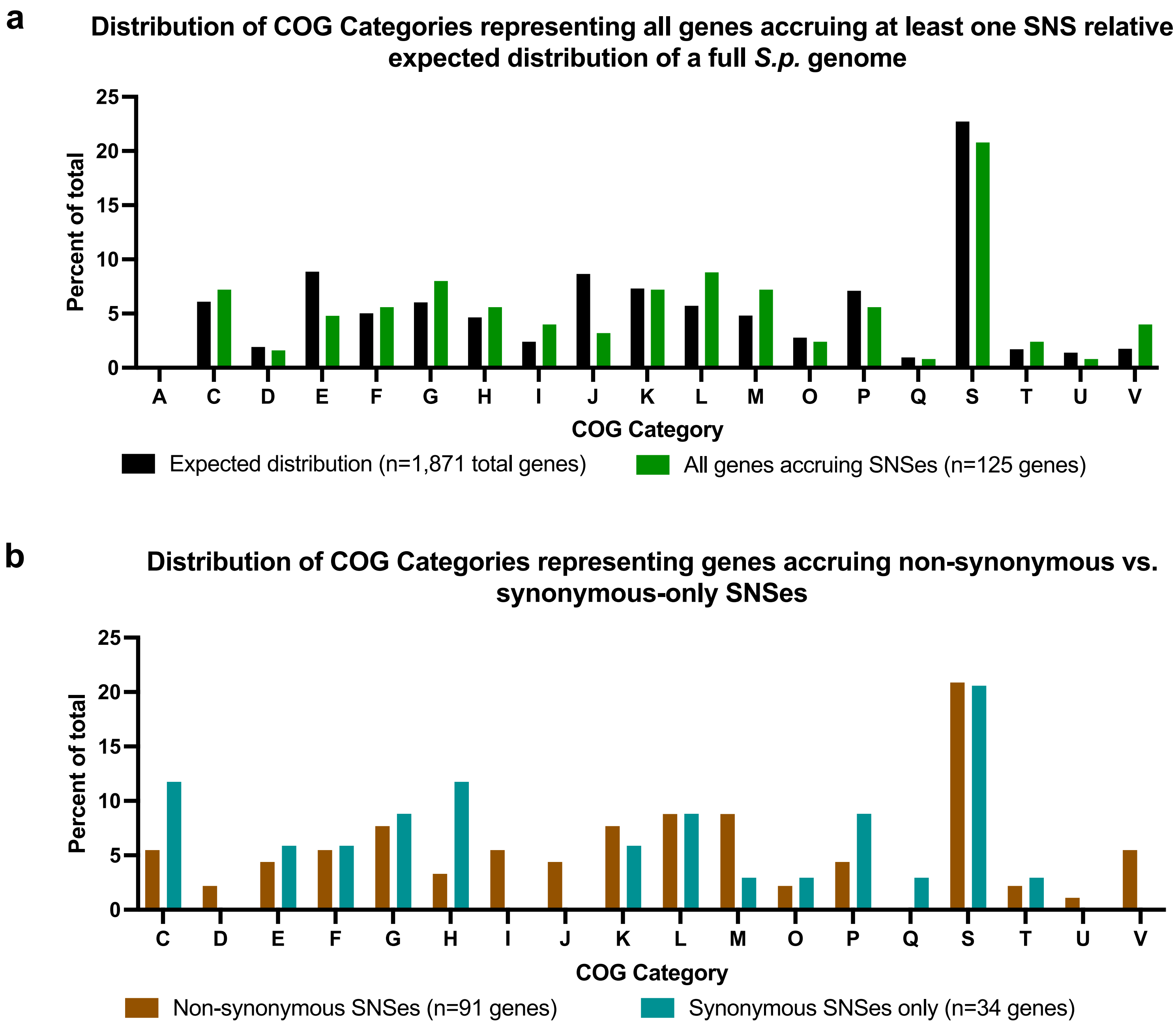

Supplementary Figure 7: COG distributions. (A) Distribution of COG categories across a representative *S. pseudintermedius* genome (SF_0321) compared to that of all genes accruing SNSes. **(B)** Distribution of COG categories across all NSS and SSO genes.
