# Supplementary material for: Diagnostic and commensal Staphylococcus pseudintermedius genomes reveal niche adaptation through parallel selection of defense mechanisms
Source: Nat Commun. 2023 Nov 3;14:7065. doi: 10.1038/s41467-023-42694-5 (PMC10624692; doi:10.1038/s41467-023-42694-5)
Supplement: Supplementary file 3 — Description of Additional Supplementary Files [file 41467_2023_42694_MOESM3_ESM.pdf]

Description of Additional Supplementary files

Supplementary Data 1: Demographic metadata for each sample and its source of isolation.

Supplementary Data 2: Aggregated demographic metadata for hosts of human colonizing and human diagnostic isolates.

Supplementary Data 3: ARG and Antibiotic susceptibility testing results.

Supplementary Data 4: Assembly quality, MLST, strain cluster, and CRISPR Cas operon data.

Supplementary Data 5: Full list of NSS and SSO genes and the strain clusters they accrue SNSes in.
