# Supplementary material for: Diagnostic and commensal Staphylococcus pseudintermedius genomes reveal niche adaptation through parallel selection of defense mechanisms
Source: Nat Commun. 2023 Nov 3;14:7065. doi: 10.1038/s41467-023-42694-5 (PMC10624692; doi:10.1038/s41467-023-42694-5)
Supplement: Supplementary file 9 — Reporting Summary [file 41467_2023_42694_MOESM9_ESM.pdf]

nature portfolio

Corresponding author(s): Gautam Dantas

Last updated by author(s): 10/1123

Reporting Summary

Nature Portfolio wishes to improve the reproducibility of the work that we publish. This form provides structure for consistency and transparency in reporting. For further information on Nature Portfolio policies, see our Editorial Policies and the Editorial Policy Checklist.

Statistics

For all statistical analyses, confirm that the following items are present in the figure legend, table legend, main text, or Methods section.

n/a	Confirmed	
☐	☒	The exact sample size (*n*) for each experimental group/condition, given as a discrete number and unit of measurement
☐	☒	A statement on whether measurements were taken from distinct samples or whether the same sample was measured repeatedly
☐	☒	The statistical test(s) used AND whether they are one- or two-sided<br>*Only common tests should be described solely by name; describe more complex techniques in the Methods section.*
☒	☐	A description of all covariates tested
☐	☒	A description of any assumptions or corrections, such as tests of normality and adjustment for multiple comparisons
☐	☒	A full description of the statistical parameters including central tendency (e.g. means) or other basic estimates (e.g. regression coefficient) AND variation (e.g. standard deviation) or associated estimates of uncertainty (e.g. confidence intervals)
☐	☒	For null hypothesis testing, the test statistic (e.g. *F*, *t*, *r*) with confidence intervals, effect sizes, degrees of freedom and *P* value noted<br>*Give P values as exact values whenever suitable.*
☒	☐	For Bayesian analysis, information on the choice of priors and Markov chain Monte Carlo settings
☒	☐	For hierarchical and complex designs, identification of the appropriate level for tests and full reporting of outcomes
☐	☒	Estimates of effect sizes (e.g. Cohen's *d*, Pearson's *r*), indicating how they were calculated

Our web collection on statistics for biologists contains articles on many of the points above.

Software and code

Policy information about availability of computer code

Data collection	No software was used for data collection.
Data analysis	All code developed for this manuscript is available at https://github.com/sanjsawhney/staphylococcus-pseudintermedius.<br><br>The following computational tools were used for this analysis: Unicycler v0.4.8 for genome assembly; seqtk v1.3 for downsampling; CheckM v1.0.13 and QUAST v4.5 for assembly quality statistics; PGAP v5.3 for gene annotation; Roary v3.12 for core gene alignment; Parsnp v1.2 for core genome alignment; mlst v2.19 for MLST typing; FastTree v2.1.10 for phylogenetic tree construction; fastANI v1.1 for ANI determination; AMRfinder v3.9.8 for antimicrobial resistance protein identification; snp-sites v2.4.0 and snippy v4.4.3 for SNP calling; Scoary v1.6.16 for genome-wide association analysis; EasyFig v2.2.2 and ApE v3.1.2 for gene structure visualization; CrisprCasFinder and clustalo v1.2.4 for CRISPR spacer identification and ANI-based spacer distance matrix construction; Bowtie2 v2.4.2, inStrain v1.5.7, and BLAST+ v2.12.0 for longitudinal SNS tracking and annotation; Prism 9 for statistical testing and data visualization.<br><br>The following R packages were used for this analysis: zipcodeR v0.3.4 for geographic distance calculations; vegan 2.5-7 for Jaccard distance calculations; ggplot2 v3.3.6, ggpubr v0.4.0, ggExtra v0.10, and pheatmap v1.0.12 for data visualization; STATS for statistical testing.

For manuscripts utilizing custom algorithms or software that are central to the research but not yet described in published literature, software must be made available to editors and reviewers. We strongly encourage code deposition in a community repository (e.g. GitHub). See the Nature Portfolio guidelines for submitting code & software for further information.

Data

Policy information about availability of data

All manuscripts must include a data availability statement. This statement should provide the following information, where applicable:

- Accession codes, unique identifiers, or web links for publicly available datasets
- A description of any restrictions on data availability
- For clinical datasets or third party data, please ensure that the statement adheres to our policy

All isolate sequencing data, including short reads and assemblies, have been deposited in the NCBI SRA and GenBank databases under BioProject PRJNA908872 [https://www.ncbi.nlm.nih.gov/bioproject/PRJNA908872/]. Specimen metadata, antibiotic susceptibility testing data, assembly quality and genome metadata, and inStrain data are provided in Supplementary Files 1, 3-5, respectively. Certain raw metadata for human-origin isolates are protected and are not available due to data privacy laws; processed data presented in aggregate are available in Supplementary File 2. Source data are provided with this paper.

Research involving human participants, their data, or biological material

Policy information about studies with human participants or human data. See also policy information about sex, gender (identity/presentation), and sexual orientation and race, ethnicity and racism.

Reporting on sex and gender	We report in aggregate the sex (biological attribute) of all humans that Staphylococcus pseudintermedius isolates were cultured from in Supplementary File 5. Sex was self-reported. Overall, 96 human diagnostic isolates were captured from female individuals, 84 human diagnostic isolates from male individuals, 14 human colonizing isolates from female individuals, and 14 human colonizing isolates from male individuals. Sex was not recorded for 2 human diagnostic isolates and 10 human colonizing isolates. Sex-based analyses of the humans contributing isolates are outside the scope of our comparative genomics analysis between isolates captured in diagnostic and colonizing niches.
Reporting on race, ethnicity, or other socially relevant groupings	We report zipcode (socially relevant grouping) of the individuals or households that Staphylococcus pseudintermedius isolates were cultured from in Supplementary File 1. Race and ethnicity were not recorded for human individuals contributing diagnostic or colonizing isolates for this study.
Population characteristics	We report age of the individuals that Staphylococcus pseudintermedius isolates were cultured from in Supplementary File 1.
Recruitment	Human clinical specimens were cultured in the Barnes-Jewish Hospital Clinical Microbiology Laboratory in St. Louis, MO according to laboratory standard operating procedures. Isolates from these specimens (n=181), primarily from human wounds, tissue, respiratory tract, and drainage, were collected as part of routine clinical care, with cultures submitted from patients with clinical symptoms suggestive of infection. Clinical specimens were plated to agar medium according to the laboratory's standard operating procedures for each specimen type. In general, specimens were plated to sheep's blood, chocolate, and MacConkey agar (Remel, Lenexa, KS). Isolates of S. pseudintermedius were most commonly obtained from the sheep's blood agar plate. All human diagnostic isolate collection occurred between December 2011 – July 2019. The diagnostic canine and feline SIG isolates were selected from a collection of isolates recovered from submissions to the Kansas State Veterinary Diagnostic laboratory. These isolates were recovered from specimens submitted for clinical culture, primarily from animal urinary tract and skin and soft tissue infections (n=100), with primary isolation occurring between January 2016 – June 2019. Isolates recovered from nondescript specimen types ("swab"), duplicate submissions from the same veterinarian-owner combination (over the entire study period), and non-viable isolates were not eligible for inclusion in this study and were replaced with the next eligible isolate until a total of 25 isolates were identified per calendar year. Colonizing isolates (from human nares, axillae, or inguinal folds and from dogs nares, mouth, or dorsal fur) and household environmental surface isolates (e.g., bed linens, kitchen table, refrigerator door handle, bathroom countertop, bathroom faucet handles, bathroom light switch, toilet seat, bathtub, television remote control, computer keyboard and mouse, and telephone) were collected through two Staphylococcus aureus surveillance projects among households with children with recent S. aureus infections through the Staph Household Intervention for Eradication (SHINE; NCT02572791) and Household Observation of MRSA in the Environment 2 (HOME2; NCT01814371) clinical trials.
Ethics oversight	Washington University Institutional review board (IRB) and Institutional Animal Care and Use Committee (IACUC) approval was obtained for this study.

Note that full information on the approval of the study protocol must also be provided in the manuscript.

Field-specific reporting

Please select the one below that is the best fit for your research. If you are not sure, read the appropriate sections before making your selection.

☒ Life sciences ☐ Behavioural & social sciences ☐ Ecological, evolutionary & environmental sciences

For a reference copy of the document with all sections, see nature.com/documents/nr-reporting-summary-flat.pdf

Life sciences study design

All studies must disclose on these points even when the disclosure is negative.

Sample size	The sample size of 501 Staphylococcus pseudintermedius genome assemblies from humans, animals, and households was determined based on availability of isolates meeting inclusion criteria.
Data exclusions	No data were excluded.
Replication	Antibiotic susceptibility testing reported in Figure 5 involved three biological replicates per isolate. All genomic data generated in this study have been deposited in NCBI, and parameters used for all computational analyses are described in the Methods; hence, replication of our outputs can be achieved.
Randomization	Randomization is not relevant as we included all available Staphylococcus pseudintermedius isolates that met inclusion criteria. "Diagnostic" isolates describe those captured on human patients in Barnes-Jewish hospital in St. Louis, MO, or canine/feline patients seen at the Kansas State Veterinary Clinic. All "commensal" isolates describe those captured on surfaces or human/pet inhabitants within participating households in the St. Louis metropolitan area.
Blinding	Blinding was not possible during initial group allocation because all samples were manually assigned their group category by the investigators through examination of sample metadata. However, blinding was not relevant in the analyses of this study as all samples were analyzed in the same manner by our computational pipeline and during phenotypic testing.

Reporting for specific materials, systems and methods

We require information from authors about some types of materials, experimental systems and methods used in many studies. Here, indicate whether each material, system or method listed is relevant to your study. If you are not sure if a list item applies to your research, read the appropriate section before selecting a response.

Materials & experimental systems

n/a	Involved in the study	
☒	☐	Antibodies
☒	☐	Eukaryotic cell lines
☒	☐	Palaeontology and archaeology
☐	☒	Animals and other organisms
☐	☒	Clinical data
☒	☐	Dual use research of concern
☒	☐	Plants

Methods

n/a	Involved in the study	
☒	☐	ChIP-seq
☒	☐	Flow cytometry
☒	☐	MRI-based neuroimaging

Animals and other research organisms

Policy information about studies involving animals; ARRIVE guidelines recommended for reporting animal research, and Sex and Gender in Research

Laboratory animals	This study did not involve laboratory animals.
Wild animals	This study did not involve wild animals. The fur of domesticated animals were swabbed at the Kansas State Veterinary Clinic ("diagnostic" isolates) or in their household of residence ("commensal" isolates). Age and species of domesticated animals that Staphylococcus pseudintermedius isolates were cultured from are reported in Supplementary File 1.
Reporting on sex	Sex of domesticated animals harboring Staphylococcus pseudintermedius isolates was not reported as it was outside the scope of a comparative genomics analysis between isolates captured in diagnostic and colonizing niches.
Field-collected samples	The study did not involve samples collected from the field.
Ethics oversight	Washington University Institutional review board (IRB) and Institutional Animal Care and Use Committee (IACUC) approval was obtained for this study.

Note that full information on the approval of the study protocol must also be provided in the manuscript.

Clinical data

Policy information about clinical studies

All manuscripts should comply with the ICMJE guidelines for publication of clinical research and a completed CONSORT checklist must be included with all submissions.

Clinical trial registration	NCT02572791, NCT01814371
Study protocol	The full study protocol is available at https://clinicaltrials.gov/study/NCT02572791 and https://clinicaltrials.gov/study/NCT01814371.
Data collection	Surfaces and inhabitants within participating households were swabbed between January 2016 and June 2019. All households are within the St. Louis metropolitan area.
Outcomes	All primary and secondary measures and assessments of those measures are available at https://clinicaltrials.gov/study/NCT02572791 and https://clinicaltrials.gov/study/NCT01814371.
